# Microstructure and Properties of Additively Manufactured AlCoCr_0.75_Cu_0.5_FeNi Multicomponent Alloy: Controlling Magnetic Properties by Laser Powder Bed Fusion via Spinodal Decomposition

**DOI:** 10.3390/ma15051801

**Published:** 2022-02-28

**Authors:** Xuan Yang, Oleg Heczko, Joonas Lehtonen, Roy Björkstrand, Mika Salmi, Volker Uhlenwinkel, Yanling Ge, Simo-Pekka Hannula

**Affiliations:** 1 Department of Chemistry and Materials Science, Aalto University School of Chemical Engineering, P.O. Box 16100, FI-00076 Espoo, Finland; joonas.m.lehtonen@aalto.fi (J.L.); yanling.ge@aalto.fi (Y.G.); simo-pekka.hannula@aalto.fi (S.-P.H.); 2 FZU—Institute of Physics of the Czech Academy of Sciences, Na Slovance 1999/2, 182 21 Prague, Czech Republic; heczko@fzu.cz; 3 Department of Mechanical Engineering, Aalto University School of Engineering, P.O. Box 14100, FI-00076 Espoo, Finland; roy.bjorkstrand@aalto.fi (R.B.); mika.salmi@aalto.fi (M.S.); 4 Leibniz Institute for Materials Engineering IWT, Badgasteiner Straße 3, 28359 Bremen, Germany; uhl@iwt.uni-bremen.de; 5 Faculty of Production Engineering, University of Bremen, Badgasteiner Straße 1, 28359 Bremen, Germany

**Keywords:** high-entropy alloys, laser powder bed fusion, selective laser melting, direct metal laser sintering, spinodal decomposition, magnetic properties

## Abstract

A non-equiatomic AlCoCr_0.75_Cu_0.5_FeNi alloy has been identified as a potential high strength alloy, whose microstructure and consequently properties can be widely varied. In this research, the phase structure, hardness, and magnetic properties of AlCoCr_0.75_Cu_0.5_FeNi alloy fabricated by laser powder bed fusion (LPBF) are investigated. The results demonstrate that laser power, scanning speed, and volumetric energy density (VED) contribute to different aspects in the formation of microstructure thus introducing alterations in the properties. Despite the different input parameters studied, all the as-built specimens exhibit the body-centered cubic (BCC) phase structure, with the homogeneous elemental distribution at the micron scale. A microhardness of up to 604.6 ± 6.8 HV0.05 is achieved owing to the rapidly solidified microstructure. Soft magnetic behavior is determined in all as-printed samples. The saturation magnetization (*M*_s_) is dependent on the degree of spinodal decomposition, i.e., the higher degree of decomposition into A2 and B2 structure results in a larger *M*_s_. The results introduce the possibility to control the degree of spinodal decomposition and thus the degree of magnetization by altering the input parameters of the LPBF process. The disclosed application potentiality of LPBF could benefit the development of new functional materials.

## 1. Introduction

Multicomponent alloys, including high-entropy alloys (HEAs), represent the fastest growing area of innovations of metallic materials since the last decade. HEAs represent not only an interesting field in materials with the diverse possibility to explore, but most importantly, the promising improvements with properties, such as, e.g., strength and ductility [1], oxidation and wear resistance [2], corrosion resistance [3], and irradiation resistance [4,5]. The concept of mixing multiple principal elements, equiatomic, or near-equiatomic compositions, in one alloy system, and resulting in the high configurational entropy to promote the solid solution, was initially materialized by Cantor et al. [6] and Yeh et al. [7]. Among varieties of HEA systems, Al*_x_*CoCrCuFeNi (*x* = 0–3, in molar ratio) has been studied by Yeh et al. [7] as an example that only simple solid solution structures form in HEAs, instead of the mixture of complicated phases produced in conventional alloys containing multiple principal elements. It has been confirmed that the Al*_x_*CoCrCuFeNi alloy consists of a single face-centered cubic (FCC) structure when the aluminum content *x* was in the range of 0 to 0.5 (equivalent to 0 to 9.09 at%), while a combination of FCC and body-centered cubic (BCC) structure was formed at *x* = 0.8. When *x* was increased further, the modulated phase structures were formed until a single BCC phase structure was achieved when *x* > 2.8 (equivalent to 35.90 at%). Apart from the microstructural evolution, the addition of aluminum in HEAs is also found to lower the density and enhance the strength as well as the oxidation resistance [8]. Therefore, AlCoCrCuFeNi-based alloys have attracted interest widely and have become one of the most studied alloy systems in HEAs [9,10,11]. To date, HEAs are mainly fabricated by arc-melting and casting, and usually with post-treatments. Furthermore, to form a single-phase structure using conventional methods, the Al content needs to be kept either quite low (below 9.09 at%) or high (above 35.90 at%). Consequently, the majority of AlCoCrCuFeNi based alloys produced conventionally display the duplex structures composed of BCC and FCC phases [12,13]. The complexity of microstructure also arises from the segregation of copper, which is presented in the AlCoCrCuFeNi based alloys [12,13]. One strategy is to reduce the content of copper. Tung et al. [12] found that a single BCC crystal phase was achieved in the as-cast AlCoCrCu_0.5_FeNi alloy, and the segregation of copper, as well as the formation of interdendrites, was suppressed significantly. On the other hand, it is reported that the single BCC phase structure has been detected in the equiatomic AlCoCrCuFeNi alloy after splat quenching or ultrarapid quenching, which involves the rapid solidification process [13,14]. However, due to the complex nature of multicomponent alloys, their fabrications challenge the controllability of techniques and the processing cost.

Laser powder bed fusion (LPBF) technique, also known as selective laser melting (SLM), is one of the most suitable additive manufacturing (AM) methods to perform the fabrication of multicomponent alloys. It exhibits high-level local controllability, high resolution and accuracy, and the capability of producing refined microstructure with superior properties owing to the rapid solidification process [15]. It exhibits the advantage of building a broad range of metal materials with complicated shapes using laser beam layer by layer, with various optional possibilities for post processing [16]. The first additively manufactured multicomponent alloy has been reported by Brif et al. [17], who fabricated FeCoCrNi alloy employing LPBF. Their results revealed promising mechanical properties comparable to conventional stainless steels, benefiting from the refinement of microstructure through the LPBF process. Some studies have investigated the printability of various AlCoCrFeNi based HEAs, including Al*_x_*CoCrFeNi [18,19], AlCoCrCuFeNi [20], AlCoCrFeMnNi [21], and AlCoCrFeNiTi_0.5_ [22]. These studies have shown that LPBF produces hierarchical microstructures, including cellular structure, modulation, and dislocation cells, which are beneficial to the advancement of mechanical properties. To date, the research concerning LPBF-built AlCoCrCuFeNi alloy remains scarce. As mentioned above, Singh et al. [13] produced an equiatomic AlCoCrCuFeNi alloy consisting of a single BCC phase by splat quenching from the melt. On the other hand, Wang et al. [20] found that the LPBF-processed equimolar AlCoCrCuFeNi alloy consists of mixed BCC and FCC phase structure with FCC content up to 40.20%. Splat quenching introduces a cooling rate of 10^6^–10^7^ K/s [13], which is similar to that in the LPBF process [23,24]. This indicates that it is crucial to optimize the process parameters in LPBF and understand the rapidly solidified microstructure, as well as its relation to properties. Such knowledge could assist the development of novel multicomponent alloys.

While the focus of multicomponent alloys research is primarily on their mechanical properties, the functional properties, such as magnetic properties, also attract interest with the promising potentials to be explored [25,26,27]. Zhang et al. [28] found that the as-cast equiatomic AlCoCrCuFeNi alloy, which consisted of a mixture of FCC and BCC phase, exhibited saturation magnetization of 38.18 Am^2^/kg at ambient temperature, which is as good as for the soft ferrite materials. The phase formation of AlCoCrCuFeNi based alloys in relation to the spinodal decomposition has been described by Singh et al. [13], Zhang et al. [29], and Peter et al. [30], as summarized in Table 1. The splat-quenched equiatomic AlCoCrCuFeNi alloy, which experienced a cooling rate of 10^6^–10^7^ K/s, showed a single BCC phase structure [13]. The moderate cooling rate of 10^3^–10^4^ K in the gas-atomized AlCoCr_0.75_Cu_0.5_FeNi powder resulted in a phase separation from solid solution into Al-Ni-rich BCC B2 structure, Fe-Cr-rich BCC A2 structure, and Cu precipitates in the nanoscale, and it was proposed that the formation of A2 structure would be suppressed by the higher cooling rate [30]. Al-Ni-rich ordered BCC phase was associated with the weakened ferromagnetism [25,31]. As the cooling rate was decreased to 10^2^–10^3^ K/s, the formation of dendritic and interdendritic features was observed at the sub-micron scale in the spray-cast sample [29]. The dendrites contained Al-Ni-rich and Fe-Cr-rich structures along with Cu-rich precipitates, and the interdendrites corresponded to Cu-rich precipitates [29]. Finally, when a cooling rate of 10–20 K/s typical for casting was employed, a complicated mixture of dendrites and interdendrites consisting of several types of BCC and FCC phase structures were observed [13]. The saturation magnetization was thereby dependent on the degree of decomposition of Cr-Fe-Co-rich phase (that existed in the dendrites) into the ferromagnetic Fe-Co-rich phase and antiferromagnetic Cr enriched phase, i.e., the higher the degree of decomposition under the slower cooling rate, the higher the reached saturation magnetization was. As a result, the as-cast sample possessed a higher saturation magnetization of 46 Am^2^/kg at 14 T and 300 K than the splat-quenched one [32]. These observations show that apart from solid solution, the spinodal decomposition is capable of improving the properties of multicomponent alloys such as magnetic properties. Such enhancement of magnetic properties by spinodal decomposition has been recently materialized by Rao et al. [33]. More importantly, according to the phase evolution stated in [13,29,30], the spinodal decomposition in the AlCoCrCuFeNi based alloy is governed by the cooling rate. This suggests that in combination with one of the advantages of LPBF, namely, controlling the cooling rate by varying the process parameters, it is feasible to govern the formation of microstructure and consequently achieve desired magnetic properties by altering the process parameters, without the need for post treatments or changing the chemical compositions of the raw powder.

The detailed microstructure of the gas-atomized AlCoCr_0.75_Cu_0.5_FeNi powder used in this research has been reported in [30,34]. This multicomponent alloy has not been fabricated by LPBF before, and the knowledge of its microstructure and properties remains inadequate. Further research is essential to evaluate especially the magnetic properties in relation to the spinodal decomposition of LPBF-built multicomponent alloys, which have been scarcely examined previously. In this study, the feasibility of additive manufacturing of AlCoCr_0.75_Cu_0.5_FeNi alloy composed of a single-phase structure and the controllability of the degree of spinodal decomposition are explored. The influence of process parameters in the LPBF process on microstructure, mechanical and magnetic properties is determined.

## 2. Materials and Methods

### 2.1. Specimens Preparation

The powder AlCoCr_0.75_Cu_0.5_FeNi was atomized with nitrogen using a free-fall atomizer in a spray forming process (ID number Sk2-800, more details as reported in [30,34]). Compared to equimolar AlCoCrCuFeNi alloy, the content of Cu is reduced in order to avoid segregation caused by its positive mixing enthalpy with each element of Cr, Co, Ni, and Fe [12].

**Figure 1:**
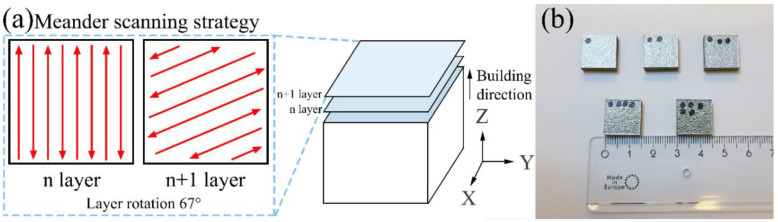
(**a**) Schematic illustration of LPBF scanning strategy, and (**b**) as-built AlCoCr_0.75_Cu_0.5_FeNi specimens applying different process parameters.

The particle class size of the raw powder used was in the range of 20–63 μm. Specimens were additively manufactured using an EOS M290 machine equipped with an Ytterbium fiber laser having 400 W maximum output and a spot size of 100 μm. Printing was carried out under a protective Ar atmosphere where oxygen content was below 0.1%. The hatch spacing was 90 μm, and the layer thickness was 20 μm. The meander scanning strategy was applied with a rotation angle of 67° between each layer, as illustrated in Figure 1a. The process parameters listed in Table 2 were selected based on the previous research reported in [20], which investigates the fabrication of equimolar AlCoCrCuFeNi alloy using LPBF, with the laser power of 160–400 W, scanning speed of 400–1600 mm/s, and corresponding volumetric energy density (VED) of 52.08–83.3 J/mm^3^. Two series of specimens with the dimensions of 15 × 15 × 3.5 mm^3^ were produced, as presented in Figure 1b. It is generally known that the key to achieving higher density and hardness is to find the optimal combination of the process parameters on which the VED depends. Accordingly, processing of the first set of samples, namely, specimens #1–3, was performed by increasing the scanning speed. The resulting variations in VED are obtained according to [35] as:

(1) $$\mathrm{VED}=\frac{P}{v\cdot h\cdot t}$$

where *P* is laser power, *v* is scanning speed, *h* is the hatch spacing, and *t* is the layer thickness. Processing of the second set of samples (specimens #3–5) was carried out applying the same VED input, but different combinations of laser power and scanning speed, for the purpose of determining the influences of these process parameters. Hatch spacing *h* and layer thickness *t* employed in all specimens remained at the unchanged value of 90 μm and 20 μm, respectively. As a reference, a pulsed electric current sintered (PECS) sample was also produced from the same set powder with a particle class size of <200 μm. The details of the fabrication and characterization are presented in the Supplementary Materials, including the Archimedes density and hardness which are given in Table S1.

### 2.2. Characterization Methods

The as-built specimens were initially ground with fine SiC papers down to P2500. The chemical composition was determined by the PANalytical Axios^mAX^ 3 kW wavelength dispersive X-ray fluorescence (WDXRF) spectrometer (PANalytical, Almelo, The Netherlands). The phase structure was identified by the PANalytical X’Pert PRO MPD diffractometer applying Co-K_α_ radiation operating at 40 kV and 40 mA, with a step size of 0.0131° and a counting time of 119.85 s per step. The phase quantification was carried out on the X-ray diffraction (XRD) pattern employing the Rietveld refinement method by X’Pert HighScore Plus software (version 4.8). The theoretical density of each specimen was calculated based on XRD patterns. The density and relative density were estimated by the Archimedes principle. The electrochemical etching was performed with the 13 vol.% HNO_3_-ethanol solution at −23 °C under 8 V. The microstructure of the specimens was studied by the JEOL JIB-4700F (JEOL, Tokyo, Japan) focused-ion-beam scanning electron microscope (FIB-SEM) equipped with a JEOL JED-2300 Analysis Station Plus energy-dispersive X-ray spectrometer (EDS) system. The preparation of EBSD specimens was performed using the Buehler VibroMet 2 vibratory polisher (Buehler, Lake Bluff, IL, USA) with Al_2_O_3_ suspension with a particle size of 0.05 μm for 18 h. The crystal orientation was characterized by the Zeiss Ultra 55 field-emission scanning electron microscope (FE-SEM) (Zeiss, Oberkochen, Germany) equipped with an Oxford Instruments Nordlys F^+^ electron backscatter diffraction (EBSD) camera. A step size of 1 μm was employed. Grain structure was determined using fast multiscale clustering (FMC) [36] using the following values: *C*_Maha_ of 1, *C*_Maha0_ of 5, gammaW of 25, alpha of 0.2, and beta of 0.3. Grain size was estimated using grain boundary images obtained through FMC analysis and calculated using the method outlined by Lehto et al. [37]. The microhardness was examined by the Innovatest Nexus 4303 Vickers hardness tester according to ISO 6507-1:2018 [38], at a force of 0.4903 N and a dwell time of 10 s. Ten points were measured for each specimen.

Magnetic properties were evaluated using a vibrating sample magnetometer (VSM) installed in the Quantum Design Physical Property Measurement System (PPMS) (Quantum Design, Inc., San Diego, CA, USA). The thermomagnetic curves, i.e., magnetization versus temperature curves, were measured in low (0.01 T) and saturation (2 T) fields in the temperature interval of 10 to 800 K in an attempt to estimate the ferromagnetic Curie point. Furthermore, the magnetic behavior of one specimen was determined in 2 T field at a temperature from 305 to 1000 K. Saturation magnetization *M*_s_ from magnetization loops was measured up to 9 T field at 10 K and 300 K. To determine magnetic coercivity, the additional measurement of magnetization loops at room temperature was performed using VSM PAR (Quantum Design, Inc., San Diego, CA, USA) with an electromagnet.

## 3. Results

### 3.1. Density and Chemical Composition

**Figure 2:**
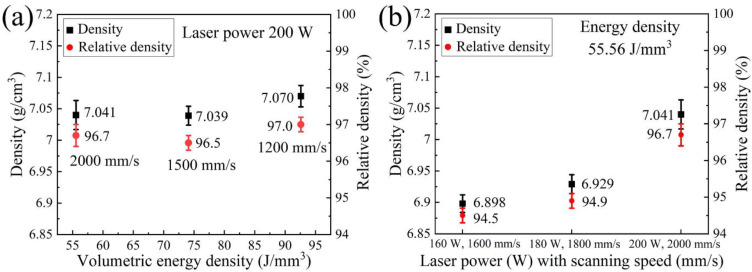
AD and RD of as-printed AlCoCr_0.75_Cu_0.5_FeNi specimens produced under (**a**) the same laser power of 200 W with different scanning speeds and (**b**) the same VED of 55.56 J/mm^3^.

The effect of process parameters on Archimedes density (AD) and relative density (RD) of LPBF-built AlCoCr_0.75_Cu_0.5_FeNi alloy is depicted in Figure 2. As shown in Figure 2a, the maximum AD of 7.070 ± 0.017 g/cm^3^ and RD of approximately 97% are achieved with the highest applied VED of 92.59 J/mm^3^. When the input VED is reduced from 92.59 to 55.56 J/mm^3^, corresponding to an increase of the scanning speed from 1200 to 2000 mm/s at a laser power of 200 W, AD and RD slightly fluctuate to 7.041 ± 0.023 g/cm^3^ and 96.7% ± 0.3%, respectively. On the other hand, in Figure 2b, both AD and RD increase noticeably from 6.898 ± 0.014 g/cm^3^ and 94.5% ± 0.2% to 7.041 ± 0.023 g/cm^3^ and 96.7% ± 0.3 % at the same applied VED of 55.56 J/mm^3^. In this case, the laser power and scanning speed are increased from 160 W and 1600 mm/s to 200 W and 2000 mm/s, respectively.

**Figure 3:**
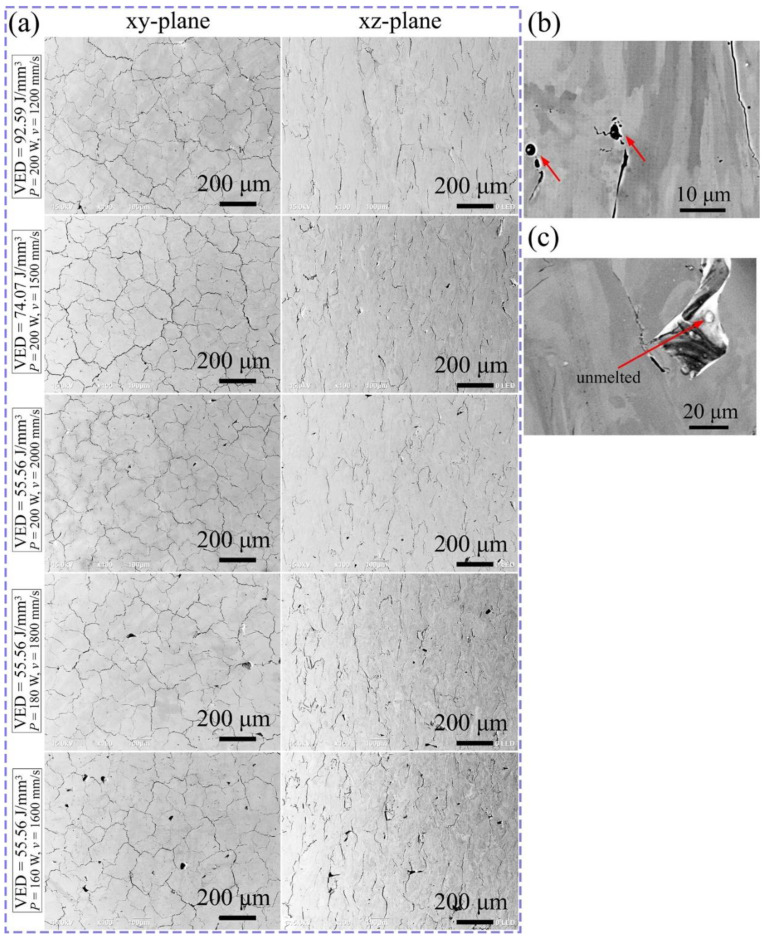
SE micrographs of as-printed AlCoCr_0.75_Cu_0.5_FeNi specimens. (**a**) Images on the xy- and xz-planes. Details of (**b**) gas pores and (**c**) lack-of-fusion defects on xz-plane.

The secondary electron (SE) images on both the xy- and xz-plane of each specimen are shown in Figure 3a. Three types of defects are found responsible for the porosity of the as-built specimens, namely, cracks, gas pores, and lack-of-fusion defects. Pores with a relatively small size and near-spherical shape are identified as gas pores (as pointed with red arrows in Figure 3b), and larger irregular-shape pores are considered to be lack-of-fusion defects [39], an example of such a defect with a nonmelted particle is displayed in Figure 3c. It can be seen that the porosity in the specimen produced with the maximum VED of 92.59 J/mm^3^ is mostly attributed to cracks and gas pores, and the cracks on the xz-plane are parallel to the building direction. The micrographs in Figure 3b,c also indicate that the cracks propagate along the grain boundaries. As the VED decreases with the growth of scanning speed, the defects are observed to become denser, and especially the lack-of-fusion defects appear. At the lowest VED input of 55.56 J/mm^3^, the incomplete melting of the powder layer is noticed more frequently as the laser power declines.

The chemical composition of as-built specimens and a reference PECS sample is presented in Table 3. It is found that among all six elements, the average content of Al changes most considerably. Comparing to the powder composition reported in [30], the composition of the reference PECS sample remains identical, while the Al content in LPBF-built specimens declines to a varying degree. This is expected, as the Al element has the lowest boiling point and heat of evaporation of the elements involved [40]. Cu content also decreases somewhat but not as clearly, whilst the content of the other four alloying elements correspondingly increases slightly. When different scanning speeds are applied at a laser power of 200 W, the element content of specimens alters only within the uncertainty of measurements despite different VEDs. Under the same VED input of 55.56 J/mm^3^, adjusted with the laser power and the scanning speed, the average content of the specimens remains roughly the same, taking into account the measurement errors. Only the average Al content appears to change slightly with changing laser power and the scanning speed. This suggests that the process parameters employed in this research reduce the Al content only marginally as neither the alteration of VED nor laser power or scanning speed affect the chemical composition of the specimens markedly.

### 3.2. Phase and Microstructure

**Figure 4:**
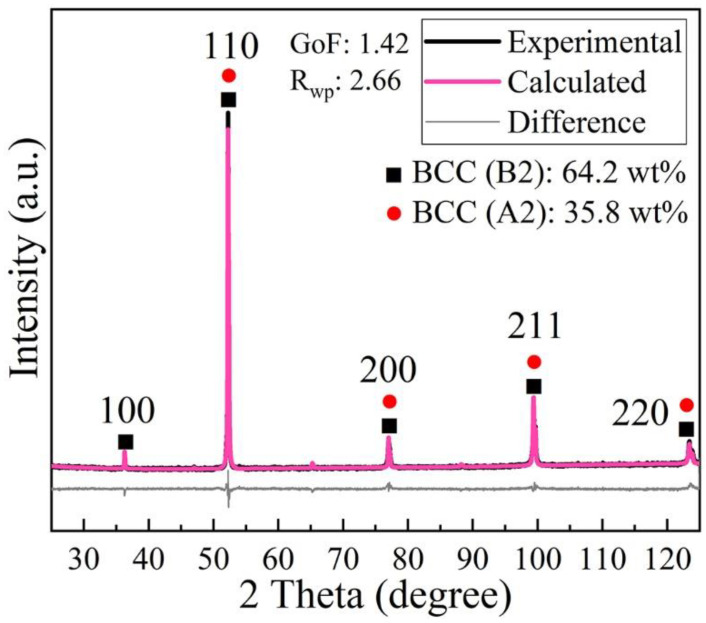
XRD experimental and refinement curves of the original powder.

Figure 4 illustrates the XRD diffraction pattern along with the Rietveld analysis of the starting powder with the particle class size of 20–63 μm. The 100 peak at 2θ = 36.28° refers to the super-lattice B2 structure. A mixture of two types of BCC phase is identified, namely, the ordered B2 phase and the disordered A2 phase. According to the phase quantification analysis carried out using the Rietveld refinement method, the lattice constant of A2 structure is estimated to be a_A2_ = 2.8732 Å, and that of B2 structure a_B2_ = 2.8744 Å. The weight fractions of A2 and B2 phase are determined to be 35.8 wt% and 64.2 wt%, respectively. These results are consistent with the observations characterized by the scanning transmission electron microscopy and atom probe tomography in [30]. It is worth mentioning that in both the previous XRD analyses on the same raw powder in [30,34], only one type of BCC phase was revealed. This is likely to result from the program settings of XRD measurement, e.g., the step size and count time per step, which are not efficient enough to depict the differences of structures.

**Figure 5:**
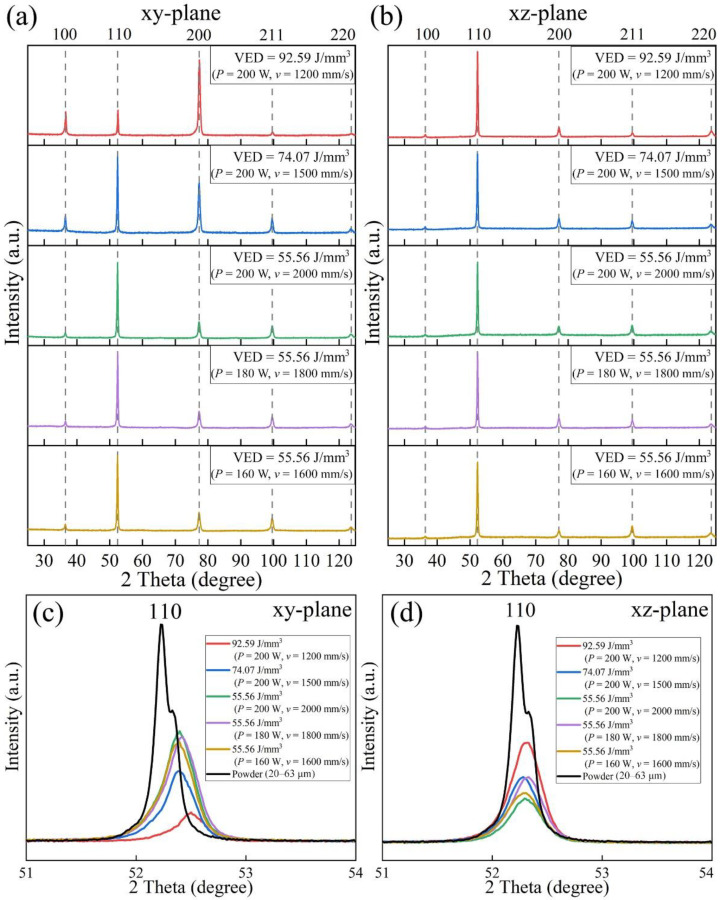
XRD patterns of as-printed AlCoCr_0.75_Cu_0.5_FeNi specimens analyzed on (**a**) xy-plane and (**b**) xz-plane, magnified 2θ of 110 peak on (**c**) xy-plane and (**d**) xz-plane.

As elongated grains are formed on the xz-plane (see in Figure 3b,c), XRD analysis has been performed on both the xy- and xz-plane of each LPBF specimen, as displayed in Figure 5. It is noticed in Figure 5a,b that only BCC phases are detected in the LPBF specimens. On the other hand, the reference PECS sample consists of both FCC and BCC phases. The weight fractions of FCC and BCC phases estimated by Rietveld refinement are 44.9 wt% and 55.1 wt%, respectively (see in Figure S1). According to Figure 5a, the intensity of the 100 and 200 diffraction peak on the xy-plane of the sample produced with the highest VED of 92.59 J/mm^3^ is enhanced in comparison with the raw powder, suggesting the presence of the preferred crystallographic texture, {100} plane is perpendicular to building direction. With the decrease of applied VED, the preferred orientation on the xy-plane gradually weakens. Under the same constant VED input of 55.56 J/mm^3^, few significant differences on the xy-plane are delineated, regardless of the alteration of both laser power and scanning speed. In addition, it is noticed that the superlattice reflection 100 is more profound on the xy-plane of samples produced by the VEDs of 92.59 and 74.07 J/mm^3^. On the xz-plane, XRD patterns of LPBF specimens appear similar to that of the starting powder, as can be seen in Figure 5b, and the differences of relative intensity on the xz-plane among each LPBF specimen are indistinguishable.

As shown in Figure 5c, the magnification of 2θ in the range of 51–54° illustrates that the 110 peaks on the xy-plane of LPBF specimens shift to a higher angle than that of the powder, which suggests that the interplanar spacing on the xy-plane of each LPBF specimen is decreased from that of starting powder. The peak on the xy-plane detected in the sample built with the largest VED of 92.59 J/mm^3^ is shifted the most. As the VED declines concomitantly with the increasing scanning speed, the degree of shift of 110 peak on the xy-plane decreases, implying that the rapid solidification-induced stress concentrations on the xy-plane are increased when the scanning speed is faster. Peaks on the xy-plane are identical at a constant VED of 55.56 J/mm^3^, suggesting that the residual stresses on the xy-plane are similar under the same VED despite the alteration in laser power and scanning speed. The magnified 2θ range from 51° to 54° in Figure 5d illustrates a smaller degree of shift but broader 110 peaks on the xz-plane when compared with the xy-plane. The broadening of XRD peaks on the xz-plane is somewhat greater than that on the xy-plane, which is likely due to the non-symmetrical cell structure and texture.

**Figure 6:**
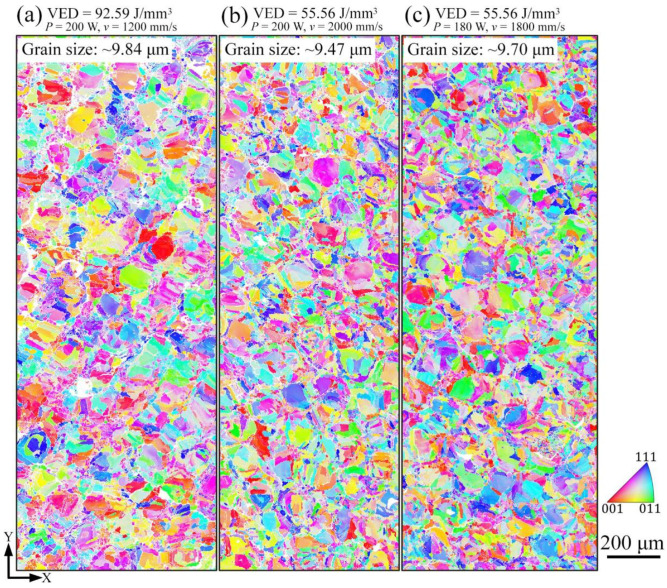
EBSD IPF maps on the xy-plane of AlCoCr_0.75_Cu_0.5_FeNi specimens produced by the different process parameters.

The EBSD analyses of inverse pole figure (IPF) maps on the xy-plane of LPBF-built AlCoCr_0.75_Cu_0.5_FeNi specimens are illustrated in Figure 6. All the specimens display the polygonal equiaxed grains on the xy-plane, and only BCC structure has been identified. The grain size of each specimen is shown on the left corner of each map. The finest grain size of ~9.47 μm is produced when the scanning speed reaches the highest value of 2000 mm/s under the laser power of 200 W, indicating that grain size increases with the decreasing applied scanning speed when the same laser power is employed.

**Figure 7:**
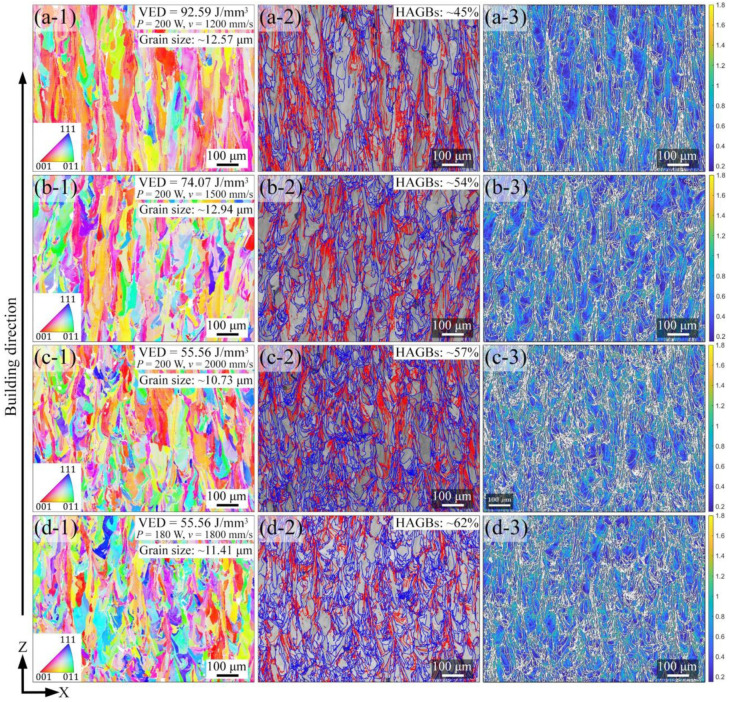
EBSD analysis on the xz-plane of AlCoCr_0.75_Cu_0.5_FeNi specimens produced by different process parameters. (**a-1**), (**a-2**), and (**a-3**) correspond to the EBSD IPF map, band contrast image with HAGBs (blue lines) and LAGBs (5–15°, red lines) map, and KAM image of the specimen produced with the VED of 92.59 J/mm^3^ (*P* = 200 W, *v* = 1200 mm/s), respectively. The rest is similar.

The EBSD IPF maps, band contrast images with grain boundary maps, and Kernel average misorientation (KAM) maps on the xz-plane of LPBF-built AlCoCr_0.75_Cu_0.5_FeNi specimens are depicted in Figure 7. The IPF maps displayed in Figure 7a-1,b-1,c-1,d-1 manifest that instead of the equiaxial grains determined on the xy-plane, the grains on the xz-plane are elongated towards the building direction, indicating that epitaxial growth occurs [41]. The average grain size of specimens estimated on the xz-plane is, therefore, larger than that determined from the xy-plane. Both the length and width of the grains reduce with the rising scanning speed; this observation is in agreement with former research, where a columnar-to-equiaxed transition (CET) of grains occurs when employing a higher cooling rate in LPBF process, and is related to the high probability of the heterogeneous nucleation under the large undercooling [42]. Merely BCC phase has also been revealed on xy-plane. The band contrast images with high angle grain boundary (HAGB, blue lines) maps and low angle grain boundary (LAGB, 5–15°, red lines) maps in Figure 7a-2,b-2,c-2,d-2 delineate the variation of the HAGBs fraction in LPBF specimens (the fraction value is shown on the upper right corner of each image). When the VED input decreases from 92.59 to 55.56 J/mm^3^, the fraction of HAGBs increases from ~45% to ~57%. It demonstrates that the specimen produced with the maximum VED contains the smallest proportion of HAGBs, as a consequence of the lower scanning speed employed. When applying the same VED of 55.56 J/mm^3^ with applied laser power of 180 W and scanning speed of 1800 mm/s, the proportion of HAGBs is determined to be ~62%, which is larger than the value of ~57% obtained with the applied laser power of 200 W and scanning speed of 2000 mm/s. The KAM maps in Figure 7a-3,b-3,c-3,d-3 quantify the average misorientation in the range of 0.2° to 1.8° within the grains. The local grain misorientation can be used to indicate the local formation of dislocation cells and dislocation density distribution in grains [43]. When the employed VED achieves the largest value of 92.59 J/mm^3^, the proportion of misorientations <0.6° within the grains is estimated to be relatively the highest. This proportion decreases as the crystal misorientation angle grows bigger when the smaller VED corresponding to a greater employed scanning speed is applied. Furthermore, under the identical input VED of 55.56 J/mm^3^, the misorientation angle decreases when the laser power and scanning speed are lowered. These results suggest that the higher cooling rate in LPBF process is responsible for the greater misorientation value in KAM map because it produces (i) higher dislocation density due to the strain localization, (ii) CET of grain structures [42], and (iii) finer grain size, which has been observed to possess a higher misorientation value [44].

**Figure 8:**
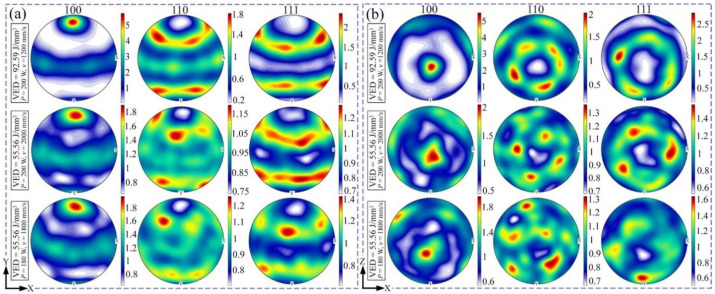
EBSD PF images of AlCoCr_0.75_Cu_0.5_FeNi specimens produced by different process parameters on (**a**) xy- and (**b**) xz-plane.

The preferred crystallographic growth orientation is represented by the EBSD pole figure (PF) images in Figure 8. The grains grow preferentially along {100} plane lies on xz-plane and parallel to building direction when the input VED is at the highest value of 92.59 J/mm^3^ (laser power of 200 W and scanning speed of 1200 mm/s). This preference weakens evidently along with the VED reducing to the lowest value of 55.56 J/mm^3^ (laser power of 200 W and scanning speed of 2000 mm/s), corresponding to the increasing of scanning speed. Under the same VED of 55.56 J/mm^3^, differences are found after the alteration of laser power and scanning speed, which indicates that texture formation does not depend entirely on the VED. The texture identified by EBSD is somewhat different from that of the XRD analysis, which is likely due to EBSD measurement being carried out on a highly localized region, where the preferred orientation alters slightly.

**Figure 9:**
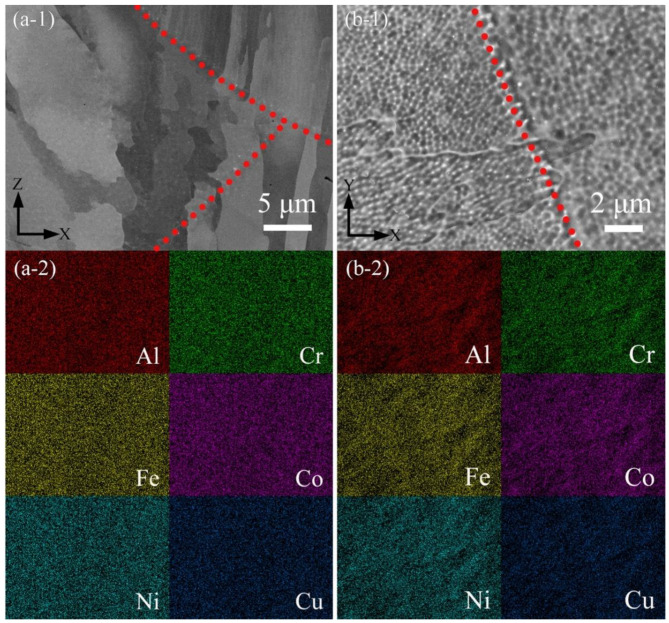
SEM observation on AlCoCr_0.75_Cu_0.5_FeNi specimen produced with the VED of 92.59 J/mm^3^ (*P* = 200 W, *v* = 1200 mm/s). (**a-1**) BSE image on the mechanical polished xz-plane with (**a-2**) corresponding quantitative EDS elemental mapping. (**b-1**) SE image on the electrochemically etched xy-plane with (**b-2**) corresponding quantitative EDS elemental distributions. Red dotted lines indicate the melt pool boundaries.

The microstructure of AlCoCr_0.75_Cu_0.5_FeNi alloy specimen produced under the VED of 92.59 J/mm^3^ (laser power of 200 W and scanning speed of 1200 mm/s) is presented in Figure 9. In the back-scattered electrons (BSE) image on the mechanically polished xz-plane shown in Figure 9a-1, the high magnification of grains and melt pool boundaries is revealed. The corresponding quantitative elemental distributions of Figure 9a-1 are illustrated in Figure 9a-2. The specimen in general has a homogeneous elemental distribution in the microscale, and no segregation is detected, even if the scanning speed was the slowest of 1200 mm/s. Typical cellular structures, as well as the boundaries of grains and melt pools on the electrochemically etched xy-plane, are shown in Figure 9b-1. It is noticed in quantitative element distribution mapping displayed in Figure 9b-2 that the elements remain homogeneously distributed, and segregation is barely observed. Due to thermal gradient, cellular structures in the area closed to the melt pool boundaries tend to be bigger in size and of irregular shape, while those located relatively far from the melt pool boundary are usually equiaxed.

**Figure 10:**
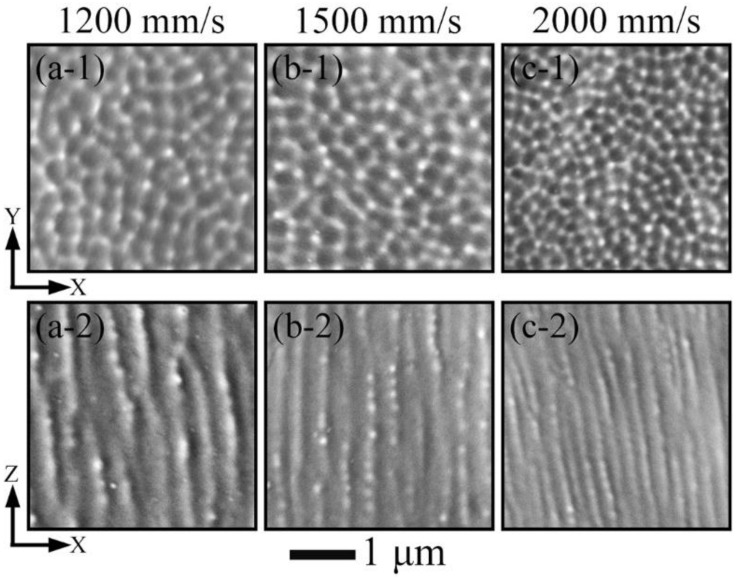
Cell structure on the xy- and xz-plane produced by laser power of 200 W applying scanning speeds of (**a-1**) and (**a-2**) 1200 mm/s, (**b-1**) and (**b-2**) 1500 mm/s, and (**c-1**) and (**c-2**) 2000 mm/s.

The variation of cell structure morphology on the xy- and xz-plane formed under the same laser power of 200 W with the alteration of scanning speed is shown in Figure 10. Similar to the observation on grains, the cells on the xy-plane incline to being equiaxial (see in Figure 10a-1,b-1,c-1), while those on the xz-plane are tended to be elongated (see in Figure 10a-2,b-2,c-2). On the xy-plane, the cell size formed under the lowest scanning speed of 1200 mm/s is estimated to be ~0.31 μm, and as the scanning speed is changed from 1500 to 2000 mm/s, the cell size is changing from ~0.28 to ~0.22 μm, respectively. This observation concurs with our previous results [45] that the cell size decreases with the growing scanning speed. The cell structures introduced by LPBF process have been found to be beneficial for enhancing the mechanical properties owing to the high degree of dislocation induced strain hardening [46]. The spacing between the cell boundaries on the xz-plane corresponds to the cell size on the xy-plane, and it alters in correlation with scanning speed as well.

### 3.3. Microhardness

**Figure 11:**
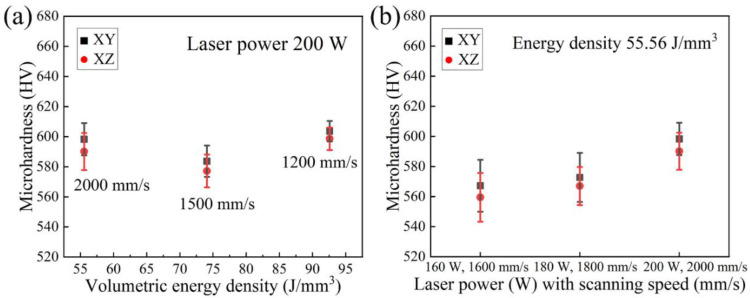
Microhardness of specimens produced under (**a**) the same laser power of 200 W with different scanning speeds and (**b**) same VED of 55.56 J/mm^3^.

**Figure 12:**
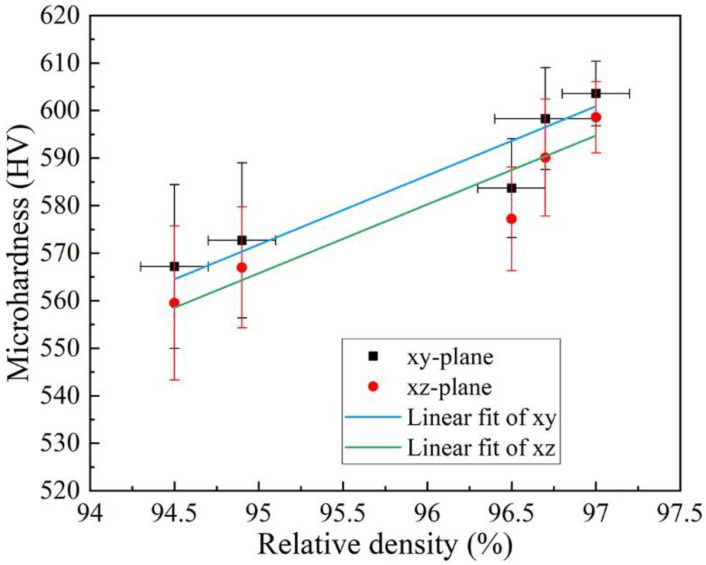
Relationship between the microhardness and relative density (RD).

The microhardness on the xy- and xz-plane of AlCoCr_0.75_Cu_0.5_FeNi specimens is presented in Figure 11. It is noticed that the microhardness of each specimen appears to be almost similar on the xy- and xz-plane, and the difference of values on the two planes of specimens is within the standard deviation of measurement even though the average microhardness value is systematically somewhat higher on the xy-plane than on xz-plane. When the VED input is reduced from 92.59 to 55.56 J/ mm^3^, maintaining constant laser power of 200 W and increasing the scanning speed from 1200 to 2000 mm/s, the hardness varies marginally (see in Figure 11a). The largest hardness 603.6 ± 6.8 HV0.05 on xy-plane and 598.6 ± 7.5 HV0.05 on xz-plane is measured from the sample produced employing 1200 mm/s scanning speed. At constant VED of 55.56 J/mm^3^, the average hardness is found to increase from 567.2 ± 17.2 to 598.3 ± 10.7 HV0.05 on the xy-plane, and from 559.5 ± 16.2 to 590.1 ± 12.3 HV0.05 on the xz-plane (see in Figure 11b) with the applied laser power and scanning speed rising from 160 W and 1600 mm/s to 200 W and 2000 mm/s, correspondingly. For comparison, the reference PECS sample exhibits a hardness of 409.5 ± 4.2 HV1. In addition, a nearly linear relation between microhardness and RD is found on both planes, as plotted in Figure 12. The microhardness improves when the LPBF sample is denser, namely, when it possesses less porosity.

### 3.4. Magnetic Properties

**Figure 13:**
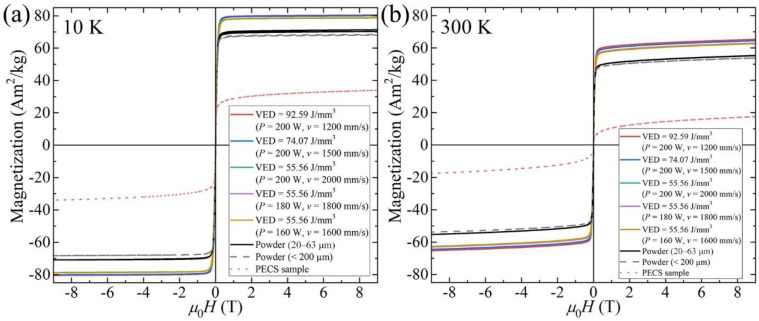
Magnetization (*M*-*H*) curves at (**a**) 10 K and (**b**) 300 K for original powder, as-built AlCoCr_0.75_Cu_0.5_FeNi specimens, and reference PECS sample.

The magnetization (*M*-*H*) curves of AlCoCr_0.75_Cu_0.5_FeNi specimens, original powder with two different particle class sizes, and one reference pulsed electric current sintered (PECS) sample, all evaluated at 10 and 300 K, are displayed in Figure 13. All samples exhibit a magnetization curve with negligible hysteresis and with no discernible magnetic anisotropy, which can be ascribed to the cubic structure of all samples. As there is no discernible effect of internal stress on magnetization, it suggests low magnetostriction. The values of saturation magnetization (*M*_s_) at 9 T of all samples are summarized in Table 4. *M*_s_ of powder with particle class sizes of 20–63 μm and <200 μm are lower than those of the LPBF specimens at temperatures of both 10 and 300 K. PECS sample exhibited surprisingly low magnetization, less than half of that of the LPBF samples, and has the lowest *M*_s_ among all measured samples. For the LPBF specimens, under the same laser power of 200 W, *M*_s_ is found to rise modestly with the decline of the scanning speed, which corresponds to the lowered cooling rate. Under the same VED of 55.56 J/mm^3^, the distinction in *M*_s_ is little among the three specimens. Identical dependence of *M*_s_ on the cooling rate is revealed at 300 K. The nearly same ratio *M*_s_ (300 K)/*M*_s_ (10 K), as well as the similar decrease of saturation magnetization, indicate a very similar saturation magnetization behavior with temperature *M*_s_ (T) and thus similar ferromagnetic Curie temperature (*T*_c_) for all LPBF samples. From the decrease of the magnetization, *T*_c_ can be estimated to be well above 400 K for LPBF samples while the PECS sample exhibited *T*_c_ of about 300 K. Coercive force of all LPBF samples is relatively low, about 760 A/m and similar to that of the starting powder (480 A/m). This indicates that internal stress has only a small effect on magnetic properties.

**Figure 14:**
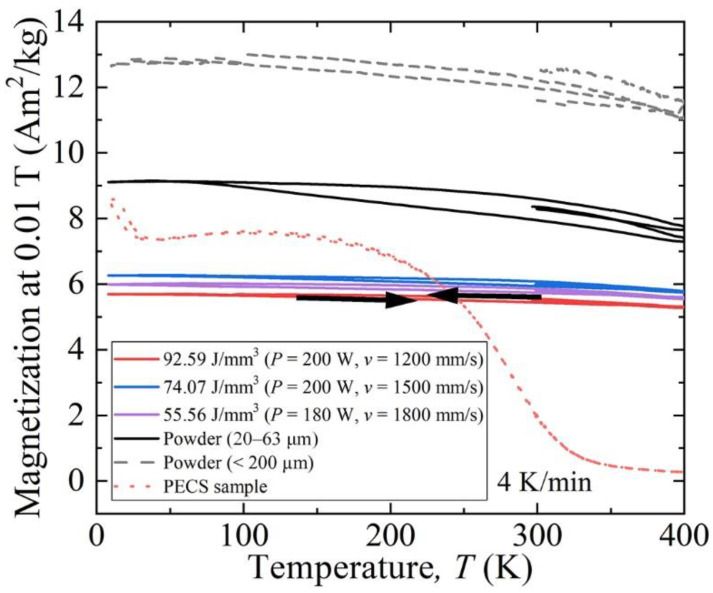
Temperature dependence of magnetization curves in the range of 10 to 400 K for original powder, as-built AlCoCr_0.75_Cu_0.5_FeNi specimens, and reference PECS sample.

Figure 14 shows the thermomagnetization (*M*-*T*) curves of LPBF specimens, raw powder, and reference PECS sample examined in a low field of 0.01 T at a temperature from 5 to 400 K. In this region, the LPBF specimens exhibit no ferromagnetic transition in contrast with the reference PECS sample, in which the Curie point is approximately 300 K. The differences in low field magnetization for powders and bulk is due to different demagnetization factor and different low field susceptibility.

**Figure 15:**
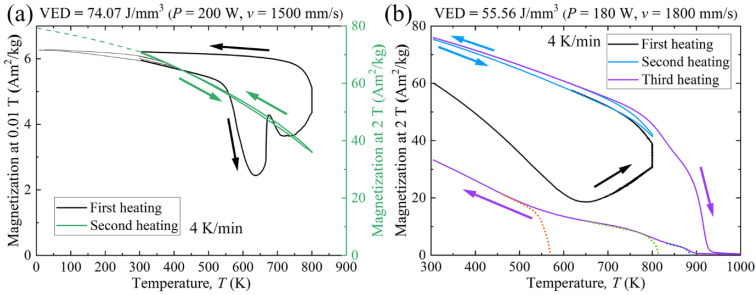
Magnetic behaviors of as-built AlCoCr_0.75_Cu_0.5_FeNi specimens. (**a**) *M*-*T* curves in the range of 10 to 800 K and (**b**) *M*-*T* curves in the range from 305 to 1000 K.

In order to determine ferromagnetic Curie point *T*_c_, one of the LPBF samples fabricated with the VED of 74.07 J/mm^3^ (laser power of 200 W and scanning speed of 1500 mm/s) was further heated in a low magnetic field (0.01 T) up to the temperature of 800 K and held there for 10 min. The evolution of the low field and saturation magnetization in the range from 10 to 800 K is shown in Figure 15a. After first heating, the *M*_s_ at 2 T and 305 K increased approximately by 10 Am^2^/kg. The magnetization did not change in the second heating in the field of 2 T. From low field magnetization (first heating round), it can be estimated that the magnetic transition occurs at the temperature of about 580 K. Further heating results in an unexpected increase and then decrease of magnetization, followed by another strong increase at 800 K. Differential scanning calorimetry (DSC) curve of spray-cast equiatomic AlCoCrCuFeNi alloy indicates no phase transition in this region [29], thus, the observed magnetization behavior indicates some magnetic transition(s) which is likely of second order. Furthermore, another sample produced by the VED of 55.56 J/mm^3^ (laser power of 180 W and scanning speed of 1800 mm/s) is heated in the field of 2 T and held at 800 K for 10 min in the first heating round, as depicted in Figure 15b. *M*_s_ is increased by nearly 15 Am^2^/kg after the first heating, and it remains almost unchanged in the second round of heating to 800 K. As the temperature further rises to 1000 K in the third heating round, the decrease of *M*_s_ is noticed. During the cooling process in the third round, three kinks are observed, as marked with dotted lines in Figure 15b, which indicates the formation of three different phases. Previously, the degeneration of spinodal decomposition structures in spray-cast equiatomic AlCoCrCuFeNi alloy was found to occur at 913 K [29]. Therefore, it is likely that the disappearance of A2 and B2 phase, as well as the formation of a new phase, is initiated after heating up to 1000 K.

## 4. Discussion

### 4.1. Effect of Process Parameters on General Microstructure

The two sets of applied process parameters suggest that laser power, scanning speed, and VED affect the specimen microstructure in different ways. Laser power is the key parameter in bringing sufficient melting of powder and governs AD and RD, and further influences mechanical properties. Scanning speed contributes also to the formation of microstructure, such as the size of cellular structure and elemental distribution. VED represents the factor that determines the combined influences of both laser power and scanning speed. For instance, both laser power and scanning speed affect the local cooling rate during the process. It is noticed that VED alone is not sufficient to explain the differences found in the current observations, such as RD, phase structure, grain and cell size, crystal misorientation, texture, and microhardness. It is in accordance with previous research [47], in which the applicability of VED confined to a narrow band has been revealed, as it fails to describe accurately the complex physics in the LPBF process. Therefore, it is proposed that all three parameters should be carefully optimized in the LPBF process. Furthermore, other parameters need to be taken into consideration in order to acquire better understanding and final properties. For instance, normalized enthalpy has been proven to be effective to determine the optimal process parameters, in which the influences of process parameters and physical properties such as thermal conductivity have been considered [48,49].

Three types of defects are identified in the as-built specimens, i.e., lack-of-fusion, spherical gas pores, and cracks. The lack-of-fusion defect occurs when the employed parameter is insufficient to fully melt the metallic powder during the process. An example of such a defect is shown earlier in Figure 3c. In addition, it is found that when the laser power reaches the largest applied value of 200 W, the lack-of-fusion defects are formed when the VED input is the lowest (55.56 J/mm^3^) and at the constant VED of 55.56 J/mm^3^, more incomplete melting defects appear when the laser power is reduced. The results suggest that both laser power and VED are required to be at a sufficiently high level to avoid the lack-of-fusion defects. The spherical gas pores, according to former research [50], can be induced by (a) the gas pores pre-existed in the gas atomized powder, (b) entrapment of the protective Ar atmosphere, or (c) the evaporation of volatile element. The most volatile element in the present work is aluminum, however, no aluminum was found in association with the pores even though the loss of aluminum in the LPBF samples was observed. Therefore, it is expected that the pores result from the protective atmosphere of either gas atomization or LPBF. This needs to be confirmed by further investigation.

The highest density is achieved in the sample produced with the maximum VED of 92.59 J/mm^3^. Most of the porosity in this sample is due to cracks, unlike those obvious lack-of-fusion defects and gas pores observed in other samples built with lower VED. The high crack sensitivity during the LPBF process has been reported in the LPBF-built equimolar AlCoCrCuFeNi HEA [20]. It is known that generally in LPBF process, internal stresses and localized strains would be created due to thermal gradient during the process [51]. In addition, the misorientation of boundaries contributes to the cracks propagating along the HAGBs instead of the LAGBs, as revealed in Figure 3 and Figure 7. This corroborates previous research, where it was described that HAGBs exhibit a higher cracking sensitivity than those of LAGBs [52,53,54]. In this research, these intergranular cracks are likely to be solidification cracks [55].

As discussed above, cracking is related to the proportion of HAGBs, and the cracks would affect the RD. The microhardness of the LPBF samples appears roughly to be a function of RD, as depicted in Figure 12, while grain size and cell size have smaller effects within the range studied. As the RD reduces, the measurement error of each hardness value varies more significantly. The highest hardness of 604.6 ± 6.8 HV0.05 on the xy-plane is achieved with the highest RD of ~97% when the HAGBs have the lowest value of ~45%. However, it is observed that the RD of each specimen produced under the laser power of 200 W with a different scanning speed of 1500 and 2000 mm/s is similar (see in Figure 2a), while the hardness of specimen produced with a scanning speed of 2000 mm/s is higher. Moreover, the RD of specimens produced under the constant VED of 55.56 J/mm^3^ with the laser power and scanning speed of 160 W and 1600 mm/s and 180 W and 1800 mm/s is noticeably lower than that of 200 W and 2000 mm/s (see in Figure 2b), but the distinction on the hardness is not as obvious (see in Figure 11b). This suggests that although the RD is relatively lower, the hardness is enhanced by the smaller cell size induced by increasing the scanning speed, as displayed in Figure 10. This is likely to be ascribed to the higher density of dislocations associated with the cells [56]. In conjunction with the controllability of the structures, especially cell size, implies the great potential of designing alloys with the desirable properties.

### 4.2. Effect of Microstructure Evolution on Magnetic Properties

The representative values of *M*_s_ of as-built LPBF specimens and raw powder are summarized in Table 5. These values were higher than those reported previously for similar compositions also highlighted in the same table. For comparison, Zhang et al. [28] obtained the *M*_s_ of 38.2 Am^2^/kg (at 1.5 T and room temperature) on as-cast AlCoCrCuFeNi alloy. The *M*_s_ of as-cast and splat-quenched AlCoCrCuFeNi alloy was disclosed by Singh et al. [32] to be 44.0 and 46.0 Am^2^/kg (at 14 T and 300 K), respectively.

The spinodal decomposition in AlCoCrCuFeNi alloy has been well established [13,29,30,32]. It has been shown that the phase decomposition already occurs at the early stage of solidification of the melt even under a high cooling rate of 10^6^–10^7^ K/s [32]. Particularly, the phase decomposition into BCC A2 and B2 structures has been observed in the gas-atomized raw powder, as shown in Figure 4. During the LPBF process, as the scanning speed varies from 2000 to 1200 mm/s under the same laser power of 200 W, accordingly, *M*_s_ increases from 78.7 to 80.5 Am^2^/kg at 10 K, and from 63.0 to 65.3 Am^2^/kg at 300 K. The differences identified in the saturation magnetization *M*_s_ of specimens (depicted in Figure 13 and summarized in Table 4) can thus be related to the dependence of *M*_s_ on the degree of spinodal decomposition, which is controlled by the cooling rate. The high cooling rate in the LPBF process leads to the suppressed decomposition, i.e., less Fe-Cr-rich A2 phase while more Al-Ni-rich B2 phase structure is produced, which causes the decrease of *M*_s_. This is in agreement with previous research [25,31], in which the Al-Ni-rich ordered BCC phase possessed weakened ferromagnetism. Singh et al. [32] showed that in the as-cast AlCoCrCuFeNi alloy aged at 873.15 K for 2 h then followed by an ice quenching, the *M*_s_ increased by 4 Am^2^/kg at 14 T and 300 K. This improvement is comparable to our results by varying the input parameters in LPBF process, as listed in Table 4. It demonstrates that LPBF provides an efficient method to enhance the magnetic properties without the need for time-consuming heat-treatment. Furthermore, it allows a local control of magnetic properties via input parameters of the LPBF process.

The variations of *M*_s_ after annealing has also been given in Table 5. Interestingly, the *M*_s_ of one LPBF specimen in this research is found to increase by approximately 15 Am^2^/kg at 2 T and 305 K after annealing at 800 K for 10 min, as displayed in Table 5. The enhanced *M*_s_ indicates that the annealing temperature around 800 K falls into the miscibility gap of spinodal decomposition, which promotes the further formation of ferromagnetic A2 structure. It suggests that if annealing this alloy at the temperature of 800 K for a suitable period, it is possible to improve the *M*_s_ significantly. As a comparison, in the research conducted by Rao et al. [33] displayed in Table 5, the Fe_15_Co_15_Ni_20_Mn_20_Cu_30_ HEA specimen was annealed at 873.15 K for 240 h to achieve an improvement from 12 to 21 Am^2^/kg at 2 T and 300 K.

When further heating up to 1000 K, the temperature is out of the range of miscibility gap, and the phase transition is also initiated. Zhang et al. [29] stated that the annealing of the spray-cast sample results in the disappearance of spinodal decomposition structures and the formation of new phases. The latter is also observed in our case (Figure 15b). Consequently, the *M*_s_ of LPBF specimen decreased while heating up to 1000 K. Moreover, it also explains the smallest *M*_s_ detected in the PECS sample. The PECS sample is sintered at the temperature of 1273.15 K, which is above the miscibility gap of spinodal decomposition, similar to the as-cast sample annealed at 1273.15 K for 2 h exhibiting the *M*_s_ of 16.1 Am^2^/kg (at 1.5 T and room temperature) [28], as displayed in Table 5. The cooling rate of 100 °C/min in the reference PECS sample is much lower than that in the LPBF process. The slow cooling process leads to the formation of dual-phase FCC + BCC structures (see in Figures S1 and S2), which is in accordance with the former investigations on AlCoCrCuFeNi based alloy produced by traditional methods with a slow cooling rate [7,9]. The BCC phase in the PECS sample is determined to be Al-Ni-rich (see in Figure S3 and Table S2). As the Al-Ni rich BCC phase discloses a weakened ferromagnetism [25,31], and furthermore, ferromagnetism is known to be sensitive to the aging temperature [57], the annealing and slow cooling are likely responsible for the smallest *M*_s_ of PECS sample (Table 4).

It can be therefore summarized that there are two stages of phase decomposition in the AlCoCrCuFeNi based alloy. When the melt solidifies at a high cooling rate, generally ≥10^3^ K/s, the phase separation into Fe-Cr-rich A2 and Al-Ni-rich B2 phase occurs. As the increase of cooling rate suppresses the formation of A2 phase, it results in the lower saturation magnetization. Once the sample is annealed at the temperature located in the miscibility gap of spinodal decomposition, further decomposition occurs, leading to the enhanced magnetization *M*_s_. At a low cooling rate, such as in a regular casting process, dendrites and interdendrites are formed, including the Cr-Fe-Co-rich regions, and if annealing the sample at a temperature fell into the miscibility gap, the Cr-Fe-Co-rich regions are separated into the ferromagnetic Fe-Co-rich structure and antiferromagnetic Cr-rich structure, and a higher degree of decomposition results in the larger *M*_s_, as disclosed by Singh et al. [32].

In this case, the magnetization measurements proved to be an efficient method to distinguish the various structural phases down to the nanoscale, which might be tedious to achieve by other characterization methods. Based on the relationship among cooling rate, degree of spinodal decomposition, and magnetization, with the great controllability of LPBF, it is feasible to design the magnetic properties as demanded.

## 5. Conclusions

To conclude, the microstructure and properties of additively manufacturing AlCoCr_0.75_Cu_0.5_FeNi alloy with LPBF process have been investigated. The feasibility of utilizing spinodal decomposition to induce the improvements of magnetic properties in multicomponent alloys is presented, and this can be easily materialized by the LPBF process.

1. Two sets of process parameters have been employed, namely, the different VEDs corresponding to the alteration of scanning speed under the same laser power, and the same VED referring to different laser power and scanning speed. The observations demonstrate that these three parameters contribute to the different aspects during the process, and they should all be optimized to achieve the best results.
2. All LPBF specimens are found to consist of BCC phase, while the reference PECS sample contains both FCC and BCC phase. The phase structure in LPBF specimens estimated by XRD reveals that the A2 structure formation is suppressed by the increasing cooling rate. Smaller cellular structures are formed by employing a higher scanning speed under the same laser power. This contributes to the high microhardness of 604.6 ± 6.8 HV0.05, despite the cracks and other defects. It is noticeably higher than 409.5 ± 4.2 HV1 of the reference PECS sample.
3. Raw powder, LPBF specimens, and PECS sample all exhibit soft magnetic behavior. The highest *M*_s_ of 65.3 Am^2^/kg is reached in as-built alloy. The saturation magnetization is related to the spinodal decomposition into A2 and B2 phase structure, and the degree of decomposition is controlled by the cooling rate, which can be adjusted by means of altering the scanning speed in the LPBF process. As the scanning speed declines, corresponding to a decreased cooling rate, a higher degree of decomposition is conducted, leading to the formation of a larger fraction of A2 structure. It contributes to the enhancement of *M*_s_ comparable to the formerly reported value of the as-annealed sample, yet without the need for time-consuming post treatment. In addition, the decomposition is furthered via annealing at 800 K for 10 min, resulting in the increase of *M*_s__,_ approximately by more than 15%.

## Figures and Tables

**Table 1:** Phase formation in correlation to fabrication method and cooling rate in AlCoCrCuFeNi based alloys.

Alloy	Method	Cooing Rate (K/s)	Phase Structure	Ref.
AlCoCrCuFeNi	Splat quenching	10^6^–10^7^	BCC	[13]
AlCoCr_0.75_Cu_0.5_FeNi	Gas atomization	10^3^–10^4^	BCC A2 + BCC B2 + Cu precipitates	[30]
AlCoCrCuFeNi	Spray casting	10^2^–10^3^	Dendrites: Al-Ni-rich and Fe-Cr-rich structures, with Cu-rich precipitates. Interdendrites: Cu-rich precipitates.	[29]
AlCoCrCuFeNi	Casting	10–20	Dendrites: Al-Ni-rich, Cr-Fe-rich, Al-Ni-Fe-rich, and Cu-rich precipitates. Interdendrites: Cu-rich.	[13]

**Table 2:** Process parameters of the LPBF-built AlCoCr_0.75_Cu_0.5_FeNi specimens.

Sample #	Power (W)	Scanning Speed (mm/s)	VED (J/mm^3^)
1	200	1200	92.59
2	200	1500	74.07
3	200	2000	55.56
4	180	1800	55.56
5	160	1600	55.56

**Table 3:** Chemical composition of as-built specimens, a reference PECS sample, and the starting powder.

Sample	Element (at%)					
	Al	Cu	Fe	Ni	Co	Cr
Powder [30]	19.5	9.57	18.8	19.5	19.6	12.9
VED = 92.59 J/mm^3^ (*P* = 200 W, *v* = 1200 mm/s)	17.8 ± 0.12	9.3 ± 0.01	19.2 ± 0.09	20.1 ± 0.05	20.5 ± 0.04	13.1 ± 0.03
VED = 74.07 J/mm^3^ (*P* = 200 W, *v* = 1500 mm/s)	17.8 ± 0.35	9.3 ± 0.02	19.2 ± 0.13	20.0 ± 0.08	20.5 ± 0.10	13.2 ± 0.08
VED = 55.56 J/mm^3^ (*P* = 200 W, *v* = 2000 mm/s)	18.1 ± 0.10	9.4 ± 0.02	19.1 ± 0.03	19.8 ± 0.07	20.3 ± 0.02	13.3 ± 0.03
VED = 55.56 J/mm^3^ (*P* = 180 W, *v* = 1800 mm/s)	17.7 ± 0.30	9.5 ± 0.05	19.1 ± 0.08	20.0 ± 0.07	20.4 ± 0.07	13.3 ± 0.08
VED = 55.56 J/mm^3^ (*P* = 160 W, *v* = 1600 mm/s)	17.6 ± 0.26	9.5 ± 0.02	19.2 ± 0.04	20.0 ± 0.10	20.4 ± 0.13	13.3 ± 0.06
PECS sample	19.3 ± 0.15	9.6 ± 0.09	18.7 ± 0.07	19.6 ± 0.06	19.9 ± 0.09	12.9 ± 0.08

**Table 4:** The saturation magnetization at 9 T of raw powder, LPBF-built AlCoCr0.75Cu0.5FeNi specimens, and reference PECS sample tested at 10 and 300 K.

Sample	*M*_s_ (Am^2^/kg)		*M*_s_ (300 K)/*M*_s_ (10 K)
	10 K	300 K	
Powder (20–63 μm)	71.3	55.3	0.78
Powder (< 200 μm)	68.4	53.8	0.79
VED = 92.59 J/mm^3^ (*P* = 200 W, *v* = 1200 mm/s)	80.5	65.3	0.81
VED = 74.07 J/mm^3^ (*P* = 200 W, *v* = 1500 mm/s)	80.1	64.4	0.80
VED = 55.56 J/mm^3^ (*P* = 200 W, *v* = 2000 mm/s)	78.7	63.0	0.80
VED = 55.56 J/mm^3^ (*P* = 180 W, *v* = 1800 mm/s)	78.9	63.0	0.80
VED = 55.56 J/mm^3^ (*P* = 160 W, *v* = 1600 mm/s)	78.9	62.6	0.79
PECS sample	33.9	17.6	0.52

**Table 5:** The saturation magnetization of multicomponent alloys produced by various methods.

Alloy	Method	*M*_s_ (Am^2^/kg)	Annealing Temperature and Time	*M*_s_ after Annealing (Am^2^/kg)	Ref.
AlCoCr_0.75_Cu_0.5_FeNi (powder, 20–63 μm)	Gas atomization	55.3 (at 9 T and 300 K)	-	-	This work
AlCoCr_0.75_Cu_0.5_FeNi (VED = 92.59 J/mm^3^ (*P* = 200 W, *v* = 1200 mm/s))	LPBF	65.3 (at 9 T and 300 K)	-	-	This work
AlCoCr_0.75_Cu_0.5_FeNi (VED = 55.56 J/mm^3^ *(P* = 180 W, *v* = 1800 mm/s))	LPBF	60.1 (at 2 T and 305 K)	800 K, 10 min	75.9 (at 2 T and 305 K)	This work
AlCoCrCuFeNi	Splat quenching	46.0 (at 14 T and 300 K)	-	-	[32]
AlCoCrCuFeNi	Casting	44.0 (at 14 T and 300 K)	-	-	[32]
AlCoCrCuFeNi	Casting	38.2 (at 1.5 T and room temperature)	1273.15 K, 2 h	16.1 (at 1.5 T and room temperature)	[28]
Fe_15_Co_15_Ni_20_Mn_20_Cu_30_	Homogenization	12.0 (at 2 T and 300 K)	873.15 K, 240 h	21.0 (at 2 T and 300 K)	[33]

## Data Availability

The raw/processed data required to reproduce these findings cannot be shared at this time, as the data also form part of an ongoing study.

## Supplementary Materials

The following supporting information can be downloaded at: https://www.mdpi.com/article/10.3390/ma15051801/s1, Figure S1: XRD experimental and refinement patterns of PECS AlCoCr_0.75_Cu_0.5_FeNi alloy; Figure S2: Secondary electron (SE) image of PECS AlCoCr_0.75_Cu_0.5_FeNi alloy; Figure S3: SEM-EDS results of PECS AlCoCr_0.75_Cu_0.5_FeNi alloy. (a) Secondary electron (SE) image. EDS spectra of (b) Point 1 and (c) Point 2 indicated in (a); Table S1: Archimedes density and Vickers hardness of PECS AlCoCr_0.75_Cu_0.5_FeNi alloy; Table S2: Chemical compositions of Point 1 (FCC phase) and Point 2 (BCC phase) displayed in Figure S3.
