# Supplementary material for: Microstructure and Properties of Additively Manufactured AlCoCr0.75Cu0.5FeNi Multicomponent Alloy: Controlling Magnetic Properties by Laser Powder Bed Fusion via Spinodal Decomposition
Source: Materials (Basel). 2022 Feb 28;15(5):1801. doi: 10.3390/ma15051801 (PMC8911743; doi:10.3390/ma15051801)
Supplement: Supplementary file 1 [file materials-15-01801-s001.zip › materials-1577330-supplementary.pdf]

materials

MDPI

Article

Microstructure and Properties of Additively Manufactured $AlCoCr_{0.75}Cu_{0.5}FeNi$ Multicomponent Alloy: Controlling Magnetic Properties by Laser Powder Bed Fusion via Spinodal Decomposition

Xuan Yang [1,*], Oleg Heczko [2], Joonas Lehtonen [1], Roy Björkstrand [3], Mika Salmi [3], Volker Uhlenwinkel [4,5], Yanling Ge [1] and Simo-Pekka Hannula [1]

Materials and Methods

The $AlCoCr_{0.75}Cu_{0.5}FeNi$ powder with a particle class size < 200 μm was employed to perform the pulsed electric current sintering (PECS) in Ar atmosphere with a cylindrical graphite die with an inner diameter of 20 mm, employing the FCT HP D 25-2 unit. The sintering was hold for 5 minutes at the temperature of 1000 ˚C and the pressure of 35 MPa, and both heating and cooling rate were kept at 100 ˚C/min. The as-sintered PECS sample was in the annealed state. The density was estimated based on Archimedes' principle. The Vickers hardness was evaluated using an Innovatest Nexus 4303 tester, with the test force of 9.807 N and dwell time of 10 s. Seven points were measured. The chemical composition was investigated using the PANalytical Axios^{mAX} 3 kW wavelength dispersive X-ray fluorescence (WDXRF) spectrometer. The X-ray diffraction pattern was identified by PANalytical X'Pert PRO MPD diffractometer with Co-K_{α} radiation under 40 kV and 40 mA. The step size was 0.0131°, and the counting time was 29 s. The Rietveld refinement method was utilized to estimate the phase quantification using X'Pert HighScore Plus software (version 4.8). The surface morphology and quantitative elemental analysis were carrying out by the TESCAN Mira3 scanning electron microscope (SEM) equipped with the Thermo Fisher Scientific energy-dispersive X-ray spectroscopy (EDS).

Supplementary Text

Table S1. Archimedes density and Vickers hardness of PECS $AlCoCr_{0.75}Cu_{0.5}FeNi$ alloy.

Sample	Archimedes Density (g/cm^3)	Vickers Hardness (HV1)
PECS $AlCoCr_{0.75}Cu_{0.5}FeNi$ alloy	7.235 ± 0.014	409.5 ± 4.2

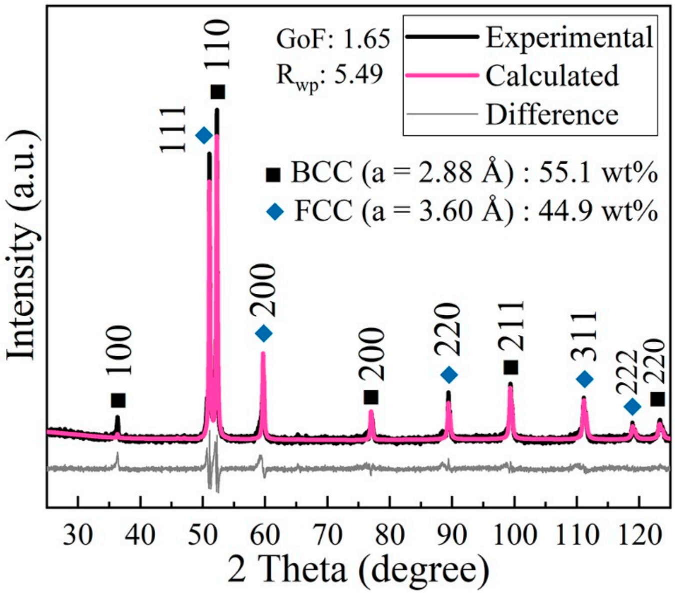

Figure S1. XRD experimental and refinement patterns of PECS $AlCoCr_{0.75}Cu_{0.5}FeNi$ alloy.

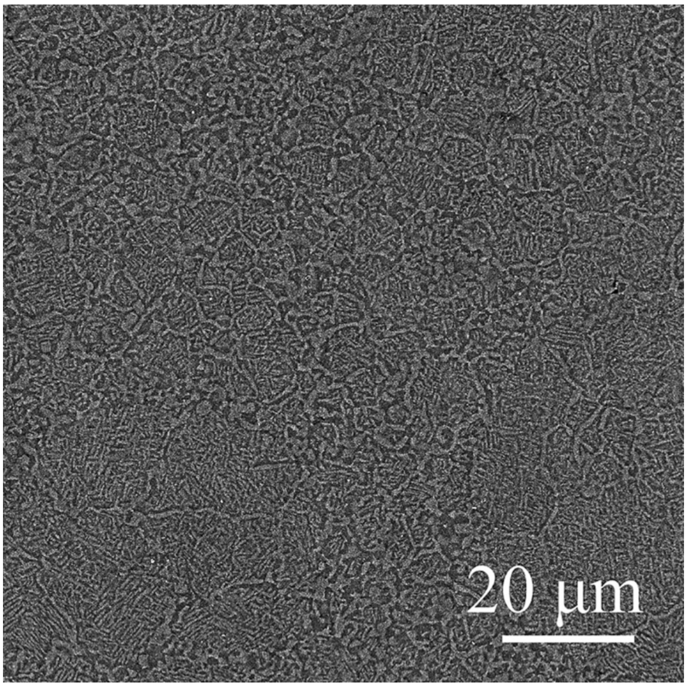

Figure S2. Secondary electron (SE) image of PECS $AlCoCr_{0.75}Cu_{0.5}FeNi$ alloy.

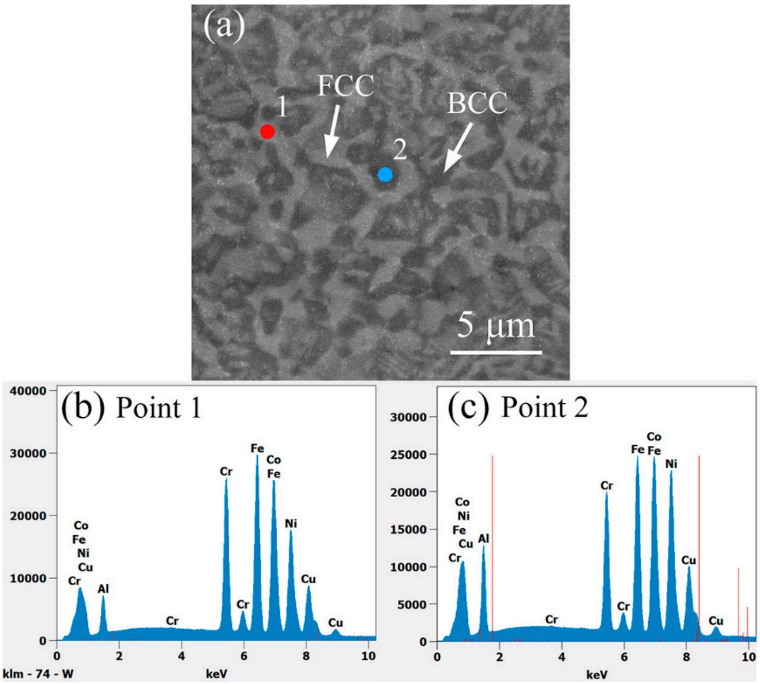

Figure S3. SEM-EDS results of PECS $AlCoCr_{0.75}Cu_{0.5}FeNi$ alloy. (**a**) Secondary electron (SE) image. EDS spectra of (**b**) Point 1 and (**c**) Point 2 indicated in (a).

Table S2. Chemical compositions of Point 1 (FCC phase) and Point 2 (BCC phase) displayed in Figure S3.

**Point**	**Element (at%)**					
	**Al**	**Cu**	**Fe**	**Ni**	**Co**	**Cr**
1	12.9	10.0	23.4	17.4	21.2	15.1
2	22.8	10.2	17.4	20.8	18.4	10.4
